# Assessing the attitude and problem-based learning in mathematics through PLS-SEM modeling

**DOI:** 10.1371/journal.pone.0266363

**Published:** 2022-05-19

**Authors:** Samina Zamir, Zhang Yang, Hao Wenwu, Uzma Sarwar

**Affiliations:** School of Education, Shaanxi Normal University, Xi’an, P.R. China; Satyawati College (Eve.), University of Delhi, INDIA

## Abstract

Mathematics plays a leading part in day-to-day life and has enhanced a necessary component for human accomplishments. Students from many countries do not reach the expected level in mathematics. Therefore, it is essential to pay close consideration to the causes related to ability in mathematics. Mathematics attitude is considered as one of the critical variables in the process of mathematics learning. This study aimed to determine students’ attitudes and achievements through problem-based learning in mathematics. The selected study group contained 600 students and 35 teachers from rural public secondary schools in District Rawalpindi, Pakistan. The data collection was done using questionnaires from students and teachers and collected data analyzed by SPSS 23 and Amos 23. This study’s result was carried out using Partial Least square structural equation Model (PLS-SEM), descriptive analysis, and hypotheses testing. The outcomes in this study indicated that the mean fluctuated between 1 to 4.5, 3.71 to 4.20, and Std. Deviation fluctuated between 0.6 to 2.0 and 0.75 to 1.55 in the students and teacher models, respectively. The results of the PLS-SEM students’ model show a negative attitude towards mathematics. The teachers’ PLS-SEM model showed the Effects of using problem-based learning (PBL) on students’ achievements. According to the hypotheses testing, the acceptance of hypotheses by stating that the Confidence in Learning Mathematics Scale (C), Value of Mathematics Scale (V), and Student Mathematics Motivation Scale (M) are significant effects for the Students’ Attitude Toward Problem-Based Learning (ATPBL). But the Attitude Toward Enjoyment in Mathematics Scale (AE) was rejected, and it did not significantly affect the ATPBL. As well as, the Problem-solving learning and students’ achievement (PLA), Advantages of problem-solving learning (APL) and Difficulties in using problem-solving learning (DPL) have a significant positive effect on the ATPBL. Finally, this study suggested that teachers also adopt new teaching methods corresponding to mathematics, and there is a need to explore particular mathematics skills to enhance students’ learning abilities.

## 1. Introduction

The most imperative things about societies are their learning ability, and learning is a necessary behaviour in life through genetic intelligence and the environment. There has been a lot of advances in educational technology in the last few decades [1]. Learning ability constantly impact the human lifestyle. As well as it is, in both developed and developing economies, entrepreneurship is considered vibrant to the nation’s competitive ability and a high-powered resource for decreasing regional inequities thereby allowing the development of the country [2,3]. According to that, the knowledge capabilities of an individual affect the student’s way of life continually [4]. Scribbling, painting and drawing perform ana significant part in the growing up of children [5,6] and it is helping to build their knowledge, personality like that. Consequently, human societies attempt to increase the method of learning in education unceasingly. As well, the ability is the core dimension of personality between learning preferences and cognitive forms. It can be defined as the preferred personal method to collect and process information, decisions, interests, ideas, and attitudes [3].

Mathematics plays a dominant role in human lives, and it is broadly applied as an essential element in personal achievements and economics [7,8]. In the 21st century, mathematics plays a significant skill in individual satisfaction and involvement in society, school, and the labour market. It appears to be a key academic filter for students’ educational trajectories [9]. Mathematics plays an essential role in supporting people to grow to reason, problem-solving skills, and thinking, and the importance of mathematics in the education system has gradually increased. Not only that, the impact of students’ mathematics achievements, including students’ ability, family socioeconomic status (SES), curriculum, many factors, peer influence, parental participation, school environment, and teachers’ quality [10,11]. By taking a positive attitude towards mathematics, students will think that mathematics is fundamental, so they try to enhance their performance in mathematics [7]. However, learning mathematics has grown into a challenge for most students today. Lack of learning despair motivates many students to say, "I am not good at mathematics", even before trying to solve mathematical problems [11]. Hence, teachers have a significant part in enhancing students’ mathematics achievement [10]. Emotional understanding, belief, and attitude are three major categories in the effective field of mathematics education [12].

However, recent worldwide determinations showed that students from many countries do not accomplish as anticipated in mathematics [13]. Therefore, one must pay close attention to the factors related to mastering mathematics and attitudes are one of the variables that can play a crucial role in learning mathematics [7].

The recent regeneration of mathematics education has brought new requirements. These provide students with meaningful activities that allow them to share their information in society. Various learning approaches focus on actions are used mainly in primary schools. One method is "problem-based learning" (PBL), which is a skill-based learning technique used to investigate and solve complicated real-life difficulties [14]. Most of the newest studies on PBL accentuate that it is a technique to enable students to enthusiastically play the part of learners. Most of the studies on PBL focus on teaching in different fields of education. These studies focus on mathematics education, science, engineering, and medicine [15]. Kaptan [16] documented that the PBL method is very important for students to improve the skills and knowledge learned in mathematics class to their daily issues and daily life. Theoretically, PBL is based on constructivism, and its instructional design method is based on problem-solving and "contextual learning" [14]. In general, PBL is considered to contribute to increasing and maintaining academic success [17] increase performance abilities [18] have a confident impact on attitudes towards classes [15], self-learning abilities and improve communication, as well as independent working abilities and motivation, and produce more reasonable explanations to problems [19]. Therefore, students’ attitudes toward mathematics have been researching worldwide for several decades [20].

## 2. Theoretical reviews

Attitude is "a learned inclination on the part of an individual to respond positively or negatively to the concept, situation, some object, or another person" [21]. Thus, the attitude towards mathematics can be an aggregation of mathematical emotions and beliefs. Allport [22] defines an attitude as "a mental or neural state of readiness, prepared over practice, applying a directive or dynamic effect upon the individuals’ feedback to all objects and circumstances with which it is associated". Adediwura [23] describes attitude as a persons’ positive, neutral or negative thinking about mathematics. A positive attitude is very instructive because research shows that there is a link between student performance and their attitude toward mathematics [24]. Students who have a positive attitude toward mathematics have better problem-solving abilities and are better able to resolve unusual difficulties [25]. They capitalize more energy in solved problems and give up when the problem cannot be solved. Attitude is also be interchanged with personality and is recognized as a multidimensional structure, including self-confidence or anxiety, such as enjoyment or not, commitment or avoidance, beliefs about whether mathematics is difficult or easy, unimportant or important, uninteresting, interesting, and useless [12]. Köğce [26] showed that the mathematics attitude is subjective in some factors, and it can be considered as several groups: firstly, reasons connected with the student, secondly, reasons associated to the teacher and school, and finally reasons related to the society and environment. Reasons related to the students’ mathematical results, their past practices [27], and social image of the mathematics. Not only that, but the reasons also related with the teachers and their content of knowledge, resources used in the classroom, the teaching methods, personality, teaching topics with real-life enriched examples [28], and the teachers’ attitude towards mathematics. Therefore, their teachers’ attitudes influence students’ attitudes [29]; teachers’ wrong beliefs about mathematics powerfully affect their teaching practices [30]. As well as it is vital to improving a positive attitude towards mathematics between students and teachers.

There have been a lot of improvements in educational technology in the last few decades [1] like online education. Students can use this technology any subject areas (especially mathematics) to improve their knowledge. But empirical studies have found that students feel that they learn better in physical classrooms than through online education [31]. Hence, Educational technology is affecting the students and teachers’ attitude toward problem-based learning mathematics.

In recent times, many researchers have pointed out the student attitude and teachers’ attitude towards problem-based learning in mathematics in several cities /countries around the world. The attitude towards mathematics has been considered for past years and shows a high relationship between attitude (including motivation, enjoyment, and self-confidence) and mathematical performance. Mezirow [32] defines learning as a cycle that starts from experience, continues to reflect, and leads to action, which becomes the experience of reflection. Valkenburg [33] found that children give their attention very rapidly to media content that was only moderately various from their existing capabilities and knowledge and teachers should give their attention for that [34]. Attitude towards mathematics is the students’ and teachers’ prepared preference to behave, perceive, feel, and think towards mathematics. Many studies have been established to assess the effect in mathematics [35].

Yılmaz [28] presented a positive and vital association between students’ attitudes towards mathematics use and mathematics accomplishment. Secondly [36], proposed a progressive connection between mathematics accomplishment and mathematics attitudes. They revealed that scholars improve attitudes, ideas, and feelings about school subjects from different sources. Thirdly, Colomeischi [37] analyzed a correlation between learning style and gender, attitude towards mathematics, and mathematical achievement. Thus, Bayaga [38] explained the students’ attitudes toward mathematics achievement using a variety of factors (attitude, mathematics self-concept, school condition, family background, teaching, and parent’s educational level) and approaches. We’ve looked into the relationship between math attitude and mathematics performance. A positive relationship between attitudes toward mathematics and academic achievement has been established in the majority of studies conducted across a range of age groups. According to some of the findings, having a negative attitude toward mathematics is associated with minor academic consequences in college students [39,40] and children [7]. However, in addition to doing so, Zsoldos-Marchis [24] investigated the problem-solving potential of various primary preschool teachers’ attitudes toward mathematics.

Moreover, Russo [41] documented the association between math teachers’ enjoyment and attitudes toward student struggle and the number of times teachers spent teaching math. There are more methods developed around the world to analyze attitudes towards mathematics. Among them, one of the most well-known analysis methods is the Partial Least Structural Equation Model (PLS-SEM), and it is a flexible modeling method without data distribution assumptions. It is also essential and suitable for various education analyses. The main aim of this study was to estimate the student attitude and teachers’ attitudes towards problem-based learning in mathematics.

## 3. Methodology

### 3.1. Participants and data collection

The study population comprised 3,300 secondary mathematics students and 35 mathematics teachers in District Rawalpindi’s 35 rural public secondary schools. The population is the mathematics students and teachers in North Punjab District Rawalpindi Government Areas as of the 2020/2021 academic session. This study selected the North Punjab district because the schools and education system are better than other areas. Moreover, belonging to the Rawalpindi district so it will be convenient to access the schools. This study selected rural schools because, in mathematics, 10^th^ class students score low compared to urban schools.

First, purposive sampling will be used in identifying and selecting schools that meet the following criteria:

- Evidence of continuous presentation of candidates for external examination in mathematics.
- Availability of qualified mathematics teachers who used the problem-based learning method in their class.
- Availability of teacher’s students and schools who agree to this study. Due to religious, cultural, regional, and local barriers.

The current study was approved by the Educational Research Ethics committee from the School of Education, Shaanxi Normal University. All procedures performed in the study involving human participants were in accordance with the ethical standards of the institutional research committee and consent was obtained from each respondent. Additional information regarding the ethical, cultural, and scientific considerations specific to inclusivity in global research is included in the Supporting Information (S1 Appendix).

By the above criteria, 35 schools will be purposively selected. In these schools, 600 mathematics (female, male) students and 35 mathematics teachers applied problem-based learning methods in their classes. The general overview of the students in the study is given in Tables 1 and 2 showed that the number of teachers in gender-wise and their qualifications.

**Table 1:** General characteristics of the students.

Gender	Age				Total
	13 years	14 years	15 years	16 years	
Girls	62	59	67	50	238
Boys	95	91	102	74	362
Total	157	150	169	124	600

**Table 2:** General characteristics of the teachers.

Qualification	Male		Female	
	Frequency	Percent	Frequency	Percent
BS	3	9	5	14
M.Phil.	5	14	8	23
M.Sc.	6	17	8	23
Total	14	40	21	60

### 3.2. Data analysis

**Fig 1:**
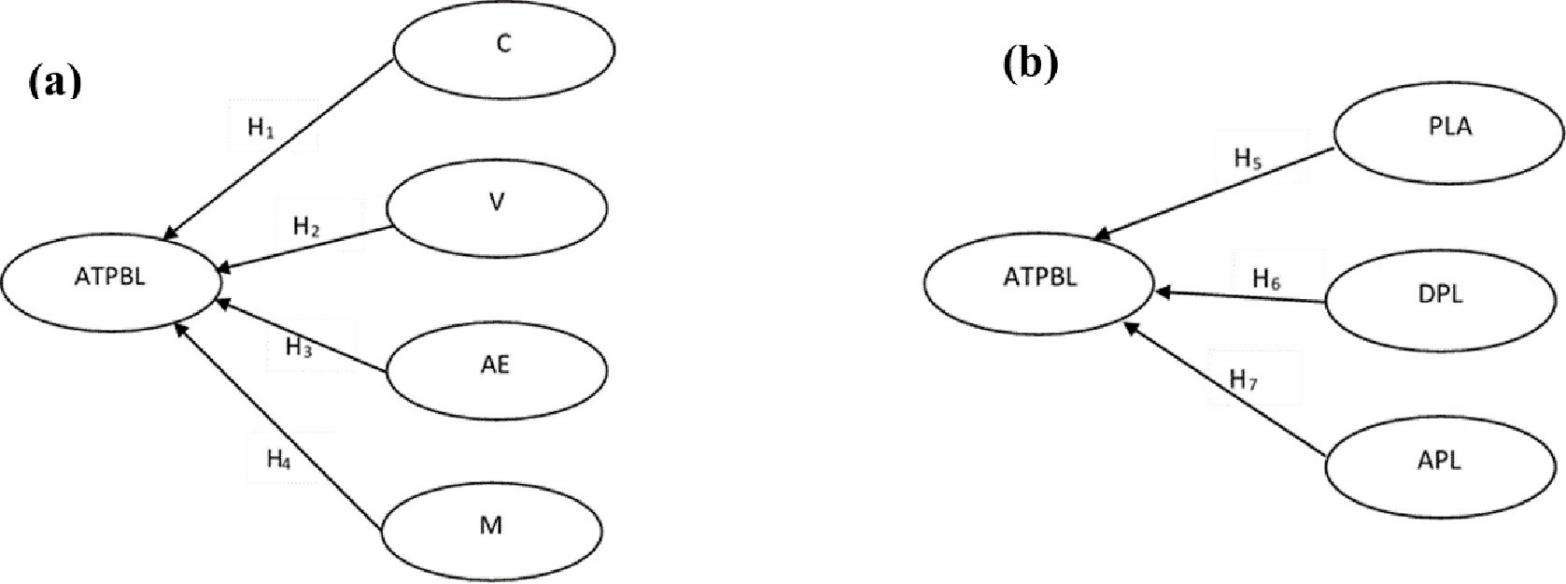
Theoretical model and hypotheses (a) students, (b) teachers.

A structured questionnaire with a Likert scale was used to investigate the mathematics attitudes toward students (see S2 Appendix) and teachers (see S3 Appendix). Descriptive statistics, hypothesis testing is used for analysis. Analysis was performed by examining the correlations, covariance patterns between the observed measures and hypotheses testing were used for this study. There are seven (7) proposed hypotheses (H_1_ to H_7_) used for analysis (see Fig 1).

**H**_**1**_: Confidence in Learning Mathematics Scale is positively influenced to Student’s Attitude Toward Problem-Based Learning.
**H**_**2**_: Value of Mathematics Scale is positively influence by Student’s Attitude Toward Problem-Based Learning.
**H**_**3**_: Attitude Toward Enjoyment in Mathematics Scale is positively influence to Student’s Attitude Toward Problem-Based Learning.
**H**_**4**_: Student Mathematics Motivation Scale is positively influencing to Student’s Attitude Toward Problem-Based Learning.
**H**_**5**_: Problem-solving learning and students’ achievements positively influence Student’s Attitude Toward Problem-Based Learning.
**H**_**6**_: Difficulties in using problem-solving learning is positively influenced Student’s Attitude Toward Problem-Based Learning.
**H**_**7**_: Advantages of problem-solving learning is positively influenced to Student’s Attitude Toward Problem-Based Learning.

Descriptive statistics are shown that provide a general overview of the data of the respondents. The collected data was analyzed by SPSS 23 version and Amos 23. For data analysis, Partial Least square structural equation Model (PLS-SEM) was used, interpreted in two stages. The first was to evaluate the student model, and the second was to assess the teachers’ model. The first (Student’s Attitude Toward Problem-Based Learning -ATPBL) model consisted of four constructs with 46 indicators—Confidence in Learning Mathematics Scale (C) = 12 indicators; Value of Mathematics Scale (V) = 12 indicators; Attitude Toward Enjoyment in Mathematics Scale (AE) = 10 indicators; and Student Mathematics Motivation Scale (M) = 12 indicators. The second model consisted of three constructs with 22 indicators—Problem-solving learning and students’ achievement (PLA) = 8 indicators, Advantages of problem-solving learning (APL) = 7 indicators, and Difficulties in using problem-solving learning (DPL) = 7 indicators can be seen in S4 Appendix.

## 4. Results and discussion

### 4.1. Student attitude towards problem-based learning in mathematics

According to Table 3, the mean fluctuated between 1 to 4.5 and Std. Deviation fluctuated between 0.6 to 2.0 and highly Std. Deviation reported from C4 (*I am always confused in my mathematics class*.) in the Confidence in Learning Mathematics Scale group. But the low value of Std. Deviation value reported from AE5 (*I really like mathematics*) in attitude toward enjoyment in mathematics scale group. Table 3 shows the results of descriptive statistics in the SEM model’s exogenous variables.

**Table 3:** The results of descriptive statistics in the SEM model’s exogenous variables.

Items	Mean	Std. Error of Mean	Std. Deviation	Variance
**C1**	3.828	0.052	1.267	1.605
**C2**	3.990	0.043	1.050	1.102
**C3**	3.925	0.055	1.349	1.819
**C4**	2.710	0.063	**1.544**	2.383
**C5**	4.270	0.037	0.912	0.832
**C6**	4.005	0.054	1.320	1.741
**C7**	3.542	0.056	1.379	1.901
**C8**	2.423	0.058	1.430	2.044
**C9**	2.152	0.057	1.391	1.935
**C10**	2.302	0.062	1.515	2.294
**C11**	2.527	0.061	1.494	2.233
**C12**	2.228	0.055	1.340	1.796
**V1**	3.788	0.053	1.299	1.686
**V2**	3.897	0.040	0.985	0.971
**V3**	3.913	0.056	1.360	1.849
**V4**	3.882	0.046	1.127	1.270
**V5**	4.183	0.037	0.895	0.801
**V6**	4.303	0.037	0.916	0.839
**V7**	4.033	0.037	0.911	0.830
**V8**	2.250	0.057	1.388	1.927
**V9**	3.800	0.050	1.228	1.509
**V10**	2.260	0.051	1.260	1.588
**V11**	1.997	0.046	1.126	1.269
**V12**	4.112	0.040	0.987	0.974
**AE1**	3.828	0.052	1.267	1.605
**AE2**	3.893	0.041	0.997	0.994
**AE3**	3.925	0.055	1.349	1.819
**AE4**	3.895	0.046	1.136	1.289
**AE5**	4.192	0.036	**0.889**	0.790
**AE6**	4.318	0.038	0.934	0.872
**AE7**	2.483	0.058	1.426	2.033
**AE8**	3.953	0.043	1.044	1.090
**AE9**	4.087	0.036	0.876	0.767
**AE10**	2.162	0.053	1.294	1.675
**M1**	3.788	0.040	0.984	0.968
**M2**	4.128	0.040	0.971	0.943
**M3**	2.363	0.056	1.370	1.878
**M4**	3.897	0.040	0.985	0.971
**M5**	3.913	0.056	1.360	1.849
**M6**	2.182	0.054	1.334	1.778
**M7**	2.555	0.060	1.476	2.177
**M8**	2.200	0.054	1.334	1.780
**M9**	2.138	0.047	1.152	1.328
**M10**	3.913	0.043	1.052	1.108
**M11**	2.545	0.059	1.438	2.068
**M12**	4.062	0.038	0.941	0.886
**ATPBL1**	3.780	0.040	0.987	0.973
**ATPBL2**	4.112	0.040	0.987	0.974
**ATPBL3**	3.788	0.040	0.984	0.968
**ATPBL4**	4.128	0.040	0.971	0.943
**ATPBL5**	3.788	0.053	1.299	1.686
**ATPBL6**	3.897	0.040	0.985	0.971

**Fig 2:**
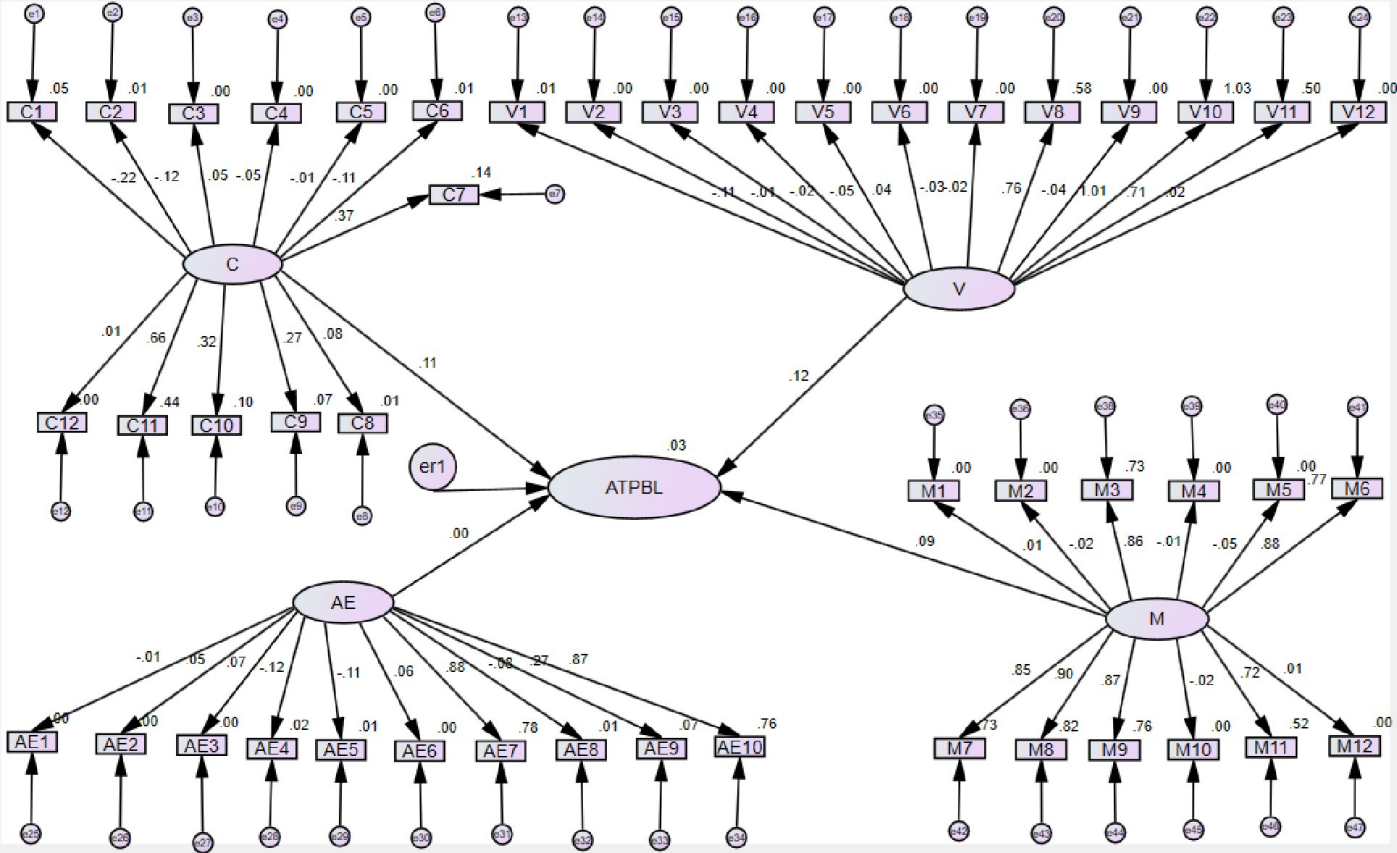
The results of structural model for PLS-SEM standardized estimation and hypotheses tests (students).

According to Fig 2, the Confidence in Learning Mathematics Scale group had a high regression weight from C11 (In terms of my adult life, it is not important for me to do well in mathematics in high school). It recorded 0.664. But in the C1 (I have a lot of self-confidence when it comes to mathematics) showed that a low regression weight. It is recorded -22. As well as Confidence in Learning Mathematics Scale and Student’s Attitude toward Problem-Based Learning presented the 0.11 Standardized Regression Weight. Value of Mathematics Scale (V) showed the high regression weight with V10 (Taking mathematics is a waste of time.), and it recorded 1.01. But in the V1 (Mathematics is a very worthwhile and necessary subject) showed a low regression weight. It is recorded -.11. As well as Value of Mathematics Scale and Student’s Attitude toward Problem-Based Learning presented the 0.12 of Standardized Regression Weight. Hence, the Attitude toward Enjoyment in Mathematics Scale (AE) had a high regression weight from AE7 (Winning a prize in mathematics would make me feel unpleasantly conspicuous), and it recorded 0.88. Hence in the AE4 (I really like mathematics.) showed a low regression weight. It is recorded -.12.

As well as Attitude toward Enjoyment in Mathematics Scale and Student’s Attitude toward Problem-Based Learning presented the 0.005 of Standardized Regression Weight. Furthermore, Student Mathematics Motivation Scale (M) had a high regression weight from M8 (The challenge of math problems does not appeal to me), and it recorded 0.90. Hence in the AE4 (I really like mathematics.) showed a low regression weight. It is recorded -.014. As well as Student Mathematics Motivation Scale and Student’s Attitude toward Problem-Based Learning presented 0.086 of Standardized Regression Weight. According to the SEM, the standardized estimation can be identified the most student have a negative attitude about mathematics. As well as Fig 2 showed that the squared multiple correlations (R^2^). A strong positive correlation was reported in V10 (Taking mathematics is a waste of time) with a 1.0 value. V2 (I want to develop my mathematical skills), V12 (I expect to have little use for mathematics when I get out of school), M1 (I like math puzzles), M2 (Mathematics is enjoyable and stimulating to me), M4 (Once I start trying to work on a math puzzle, I find it hard to stop), M12 (I do as a little work in math as possible), C5 (I learn mathematics easily.), C12 (When I hear the word mathematics, I have a feeling of dislike.), AE1 (I have usually enjoyed studying mathematics in school.) showed that, the no correlation. Not only that, but there was also no negative correlation reported in this model.

### 4.2. Hypotheses testing for Student attitude towards problem-based learning in mathematics

The proposed hypotheses of this study were tested through the standardized coefficient values and p-values in AMOS 23.0. The students’ model’s dependent variable was the Student’s Attitude Toward Problem-Based Learning (ATPBL). The Confidence in Learning Mathematics Scale (C), Value of Mathematics Scale (V), Attitude Toward Enjoyment in Mathematics Scale (AE), and Student Mathematics Motivation Scale (M) were independent variables.

Table 4 showed the acceptance of hypothesizes states that the Confidence in Learning Mathematics Scale (C), Value of Mathematics Scale (V), and Student Mathematics Motivation Scale (M) and these states are significant effects on the Student’s Attitude Toward Problem-Based Learning. But hypotheses state that the Attitude Toward Enjoyment in Mathematics Scale (AE) was rejected, and it did not significantly impact the Student’s Attitude Toward Problem-Based Learning (ATPBL).

**Table 4:** Hypotheses testing results.

Hypothesis	Hypotheses paths	Standard coefficients	P-values	Findings
H_1_	C →ATPBL	0.11	0.001	Accept
H_2_	V →ATPBL	0.12	0.001	Accept
H_3_	AE → ATPBL	0.00	0.584	Reject
H_4_	M → ATPBL	0.09	0.001	Accept

### 4.3. Effects of using Problem-based Learning (PBL) on student’s achievements

According to Table 5, the mean fluctuated between 3.71 to 4.20 and Std. Deviation fluctuated between 0.75 to 1.55 and high std. Deviation reported from PLA3 (*When I use this method*, *student achievement is high*.) in Problem solving learning and students’ achievement group. But the low value of std. Deviation value reported from APL7 (*Problem-solving reduces the need to revise prior to examinations*.) in Advantages of the problem-solving learning group.

**Table 5:** Descriptive statistics’ results in SEM model’s exogenous variables.

Items	Mean	Std. Error of Mean	Std. Deviation	Variance
PLA1	4.20	0.18	1.05	1.11
PLA2	4.31	0.19	1.11	1.22
PLA3	4.06	0.26	**1.55**	2.41
PLA4	3.97	0.21	1.22	1.50
PLA5	4.17	0.18	1.07	1.15
PLA6	3.94	0.22	1.33	1.76
PLA7	3.97	0.21	1.22	1.50
PLA8	4.11	0.18	1.08	1.16
APL1	3.74	0.24	1.42	2.02
APL2	3.91	0.20	1.17	1.37
APL3	4.14	0.16	0.94	0.89
APL4	3.71	0.24	1.43	2.03
APL5	3.80	0.26	1.51	2.28
APL6	3.71	0.21	1.23	1.50
APL7	4.29	0.13	0.75	0.56
DPL1	3.91	0.16	0.95	0.90
DPL2	4.11	0.19	1.11	1.22
DPL3	3.97	0.15	0.89	0.79
DPL4	4.06	0.15	0.91	0.82
DPL5	3.94	0.17	1.03	1.06
DPL6	3.89	0.17	0.99	0.99
DPL7	4.06	0.16	0.94	0.88

**Fig 3:**
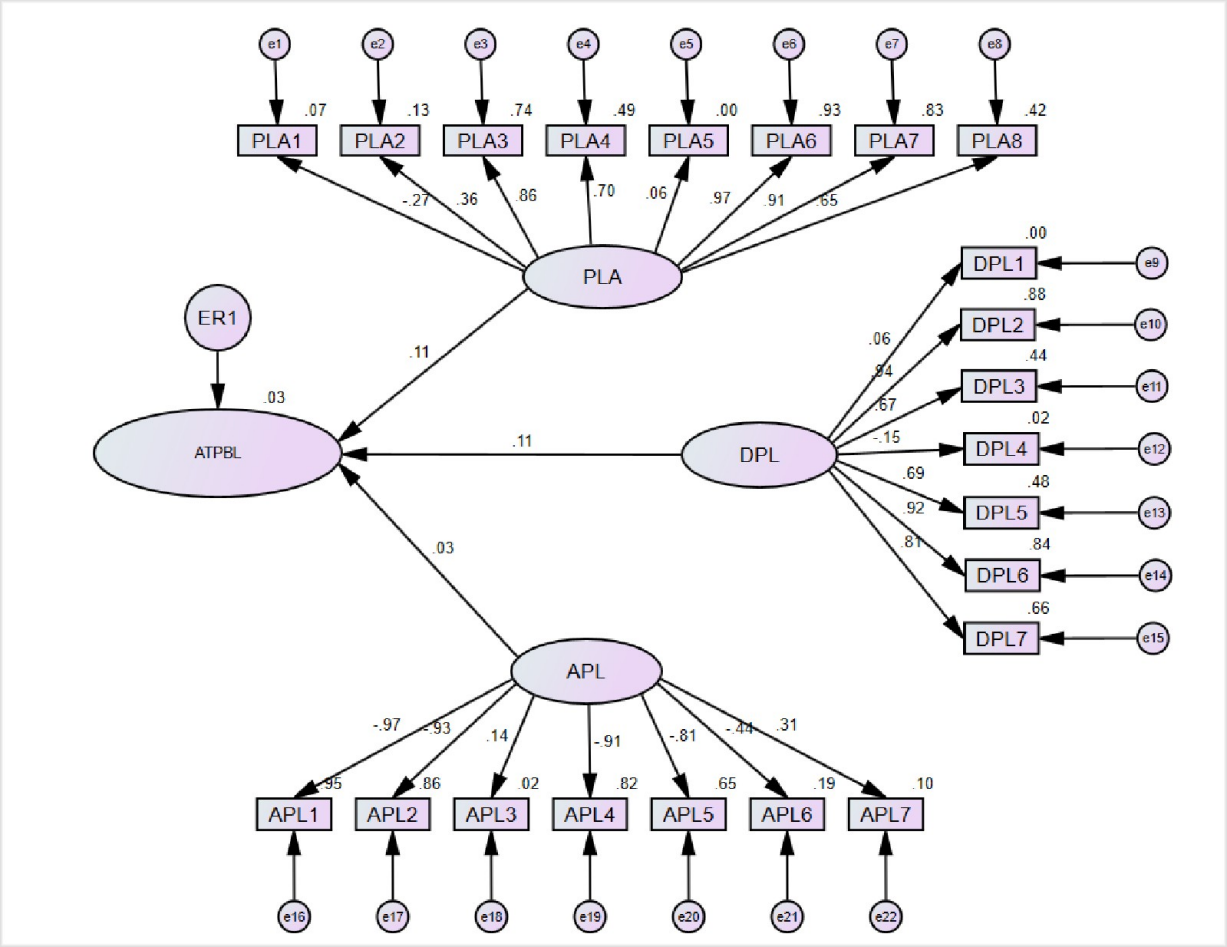
The Results of structural model for PLS-SEM standardized estimation and hypotheses tests (teachers).

According to Fig 3, the problem-solving learning and students’ achievement group had the high regression weight from PLA6 (*The mathematics curriculum is designed to use the problem-solving method frequently*.), and it recorded 0.97. However, the PLA1 (*You always get a good response from students who are motivated actively to solve the problems by themselves*.*)* showed a low regression weight. It is recorded -.269. As well as problem-solving learning and students’ achievement (PLA) and Student’s Attitude toward Problem-Based Learning presented the 0.106 Standardized Regression Weight. Advantages of problem-solving learning (APL) showed the high regression weight with APL7 (*Textbooks are structured to support problem-solving strategies*) and recorded 0.314. But in the APL1 (*Problem-solving helps students to use mathematics in their daily life*.) showed a low regression weight. It is recorded -.0.974. As well as Advantages of problem-solving learning (APL) and Student’s Attitude toward Problem-Based Learning presented the 0.031 of Standardized Regression Weight. Hence, Difficulties in using problem-solving learning (DPL) had a high regression weight from DPL2 (*This method is not suitable when the time span is short for teaching*.*)*, and it recorded 0.94. Moreover, the DPL4 (You need enough space, resources, and feasible environment in the class.) showed the low regression weight. It is recorded -.153. As well as Difficulties in using problem-solving learning (DPL) and Student’s Attitude toward Problem-Based Learning presented the 0.11 of Standardized Regression Weight.

As well as Fig 3 showed the squared multiple correlations (R^2^) and strong positive correlation reported in APL1 (*You always get a good response from students are motivated actively to solve the problems by themselves*.), DPL2 (*This method is not suitable when time span is short for teaching*.), APL2 (*You find the problem-solving method supportive for learners of all abilities in the class*.), DPL6 (*It is more difficult to satisfy slow and weak learners through problem solving*.), APL4 (*Students learn to draw diagram and pictures themselves to solve problems*.), with .949, .883, .883, .859, .844 .824, respectively. ATPBL (*Student’s Attitude toward Problem-Based Learning*), APL3 (*When I use this method*, *student achievement is high*.) PLA5 (*Problem-solving is helpful to make a learner more skilled and confident*.*)* DPL1 (*This method is difficult when students are larger in number in the classroom*.) showed the very week but positive correlation. Not only that, but there was also no negative correlation reported in this model.

### 4.4. Hypotheses testing for effects of using Problem-based Learning (PBL) on student’s achievements

The proposed hypotheses of this study were tested through the standardized coefficient values, and p-values in AMOS 23.0 for the teachers’ model. In the teachers’ model dependent variable was the Student’s Attitude Toward Problem-Based Learning (ATPBL). The Problem-solving learning and students’ achievement (PLA) Advantages of problem-solving learning (APL) and Difficulties in using problem-solving learning (DPL) were independent variables in this study.

Table 6 showed the acceptance of hypothesizes by stating that the Problem-solving learning and students’ achievement (PLA), Advantages of problem-solving learning (APL), and Difficulties in using problem-solving learning (DPL). These states have a significant positive impact on the Student’s Attitude Toward Problem-Based Learning (ATPBL).

**Table 6:** Hypotheses testing results.

Hypothesis	Hypotheses paths	Standard coefficients	P-values	Findings
H_5_	PLA →ATPBL	0.11	0.001	Accept
H_6_	DPL→ ATPBL	0.11	0.001	Accept
H_7_	APL →ATPBL	0.03	0.001	Accept

## 5. Conclusion

Information about students’ attitudes towards problem-based learning in mathematics is influential to both the students and the teachers [30]. The current study Partial Least Structural Equation Model (PLS-SEM) approach investigates the student attitude and teachers’ attitude towards problem-based learning in mathematics. The demographic data of this study have also exposed those 600 students and 36 teachers are competent in handling mathematics.

In firstly, this study estimated the student attitude towards problem-based learning in mathematics. The PLS-SEM model showed that the mean fluctuated between 1 to 4.5 and Std. Deviation fluctuated between 0.6 to 2.0. Among the 46 indicators, the C4 (I am always confused in my mathematics class) showed a high Std. Deviation and, but the low value of Std. Deviation value reported from AE5 (I really like mathematics) indicator.

According to the regression weight, in the students’ model, the high weight record in C11 (In terms of my adult life it is not important for me to do well in mathematics in high school.) and it recorded 0.664, V10 (Taking mathematics is a waste of time.). It recorded 1.01, AE7 (Winning a prize in mathematics would make me feel unpleasantly conspicuous) and it recorded 0.88, M8 (The challenge of math problems does not appeal to me), and it recorded 0.90.

According to the regression weight, in the teachers’ model, the high weight record PLA6 (The mathematics curriculum is designed to use the problem-solving method frequently) and is recorded 0.97. APL7 (Textbooks are structured to support problem-solving strategies.), and it recorded 0.314. DPL2 (This method is not suitable when time span is short for teaching.), and it recorded 0.94.

According to the hypothesizes testing, the acceptance of hypothesizes by stating that the Confidence in Learning Mathematics Scale (C), Value of Mathematics Scale (V), and Student Mathematics Motivation Scale (M) and these states are significant effects on the Students’ Attitude Toward Problem-Based Learning. But it hypothesizes by stating that the Attitude Toward Enjoyment in Mathematics Scale (AE) was rejected, and it did not significantly affect the Students’ Attitude Toward Problem-Based Learning (ATPBL). As well as the acceptance of hypothesizes by stating that the Problem-solving learning and students’ achievement (PLA), Advantages of problem-solving learning (APL) and Difficulties in using problem-solving learning (DPL) has a significant positive impact on the Students’ Attitude Toward Problem-Based Learning (ATPBL).

This study significantly revealed students’ attitudes towards mathematics and the attitudes of teachers who use it to teach mathematics. Finally, this study suggested that teachers should also adopt new teaching methods corresponding to mathematics. There is a need to explore particular mathematics skills to enhance students’ learning abilities.

## Supporting information

S1 Appendix This appendix contains Inclusivity in global research. (DOCX)

S2 Appendix This appendix contains students’ questionnaire. (DOCX)

S3 Appendix This appendix contains teachers’ questionnaire. (XLSX)

S4 Appendix This appendix contains variables. (DOCX)

S1 Dataset (RAR)
