# Supplementary material for: Assessing the attitude and problem-based learning in mathematics through PLS-SEM modeling
Source: PLoS One. 2022 May 19;17(5):e0266363. doi: 10.1371/journal.pone.0266363 (PMC9119563; doi:10.1371/journal.pone.0266363)
Supplement: S1 Appendix — (DOCX) [file pone.0266363.s001.docx]

**Study on Assessing the Attitude and Problem-Based Learning in Mathematics in Secondary Schools in Punjab, Pakistan**

**(Student’s Questionnaire)**

| School | | --------------------- | Gender | | --------------------- | | | | |
| --- | --- | --- | --- | --- | --- | --- | --- | --- | --- |
| Class | | --------------------- | Age | | --------------------- | | | | |
| **What is the influence of teaching mathematics problem-based learning on secondary school student’s attitude in mathematics?** Please rate them according to given scale.  SD= Strongly disagree, D= Disagree, U= Uncertain, A= Agree, SA= Strongly agree | | | | | | | | | |
| **Statements** | | | | **Scale** | | | | | |
| **Confidence in Learning Mathematics Scale (C)** | | | | **SD** | | **D** | **U** | **A** | **SA** |
| **+** | I have a lot of self-confidence when it comes to mathematics | | | --- | | --- | --- | --- | --- |
| **+** | I am able to solve mathematics problems without too much difficulty. | | | --- | | --- | --- | --- | --- |
| **+** | I expect to do fairly well in any mathematics class I take. | | | --- | | --- | --- | --- | --- |
| **_** | I am always confused in my mathematics class. | | | --- | | --- | --- | --- | --- |
| **+** | I learn mathematics easily. | | | --- | | --- | --- | --- | --- |
| **+** | I believe I am good at solving mathematics problems. | | | --- | | --- | --- | --- | --- |
| **+** | Mathematics does not scare me at all. | | | --- | | --- | --- | --- | --- |
| **-** | Mathematics is one of my most dreaded subjects. | | | --- | | --- | --- | --- | --- |
| **-** | My mind goes blank and I am unable to think clearly when working with mathematics. | | | --- | | --- | --- | --- | --- |
| **-** | Studying mathematics makes me feel nervous. | | | --- | | --- | --- | --- | --- |
| **-** | Mathematics makes me feel uncomfortable. | | | --- | | --- | --- | --- | --- |
| **-** | When I hear the word mathematics, I have a feeling of dislike. | | | --- | | --- | --- | --- | --- |
| **Value of Mathematics Scale (V)** | | | | | | | | | |
| **+** | Mathematics is a very worthwhile and necessary subject | | | --- | | --- | --- | --- | --- |
| **+** | I want to develop my mathematical skills | | | --- | | --- | --- | --- | --- |
| **+** | Mathematics helps develop the mind and teaches a person to think. | | | --- | | --- | --- | --- | --- |
| **+** | Mathematics is important in everyday life. | | | --- | | --- | --- | --- | --- |
| **+** | Mathematics is one of the most important subjects to study. | | | --- | | --- | --- | --- | --- |
| **+** | High school mathematics courses would be very helpful no matter what I decide to study. | | | --- | | --- | --- | --- | --- |
| **+** | I can think of many ways that I use mathematics outside of school. | | | --- | | --- | --- | --- | --- |
| **-** | Mathematics will not be important to me in my life's work. | | | --- | | --- | --- | --- | --- |
| **+** | I see mathematics as a subject I will rarely use in my daily life as an adult | | | --- | | --- | --- | --- | --- |
| **-** | Taking mathematics is a waste of time. | | | --- | | --- | --- | --- | --- |
| **-** | In terms of my adult life it is not important for me to do well in mathematics in high school. | | | --- | | --- | --- | --- | --- |
| **-** | I expect to have little use for mathematics when I get out of school. | | | --- | | --- | --- | --- | --- |
| **Attitude Toward Enjoyment in Mathematics Scale (AE)** | | | | | | | | | |
| **+** | I have usually enjoyed studying mathematics in school. | | | --- | | --- | --- | --- | --- |
| **+** | I like to solve new problems in mathematics. | | | --- | | --- | --- | --- | --- |
| **+** | I would prefer to do an assignment in mathematics than to write an essay. | | | --- | | --- | --- | --- | --- |
| **+** | I really like mathematics. | | | --- | | --- | --- | --- | --- |
| **+** | I am happier in a mathematics class than in any other class. | | | --- | | --- | --- | --- | --- |
| **+** | Mathematics is a very interesting subject | | | --- | | --- | --- | --- | --- |
| **+** | Winning a prize in mathematics would make me feel unpleasantly conspicuous | | | --- | | --- | --- | --- | --- |
| **+** | I am comfortable expressing my own ideas on how to look for solutions to a difficult problem in mathematics. | | | --- | | --- | --- | --- | --- |
| **+** | I am comfortable answering questions in mathematics class. | | | --- | | --- | --- | --- | --- |
| **_** | Mathematics is dull and boring. | | | --- | | --- | --- | --- | --- |
| **Student Mathematics Motivation Scale (M)** | | | | | | | | | |
| **+** | I like math puzzles | | | --- | | --- | --- | --- | --- |
| **+** | Mathematics is enjoyable and stimulating to me | | | --- | | --- | --- | --- | --- |
| **+** | When a math problem arises that I can’t immediately solve, I stick with it until I have solution | | | --- | | --- | --- | --- | --- |
| **+** | Once I start trying to work on a math puzzle, I find it hard to stop | | | --- | | --- | --- | --- | --- |
| **+** | When a question is left unanswered in math class, I continue to think about it afterward | | | --- | | --- | --- | --- | --- |
| **+** | I am challenged by math problems I can’t understand immediately | | | --- | | --- | --- | --- | --- |
| **_** | Figuring out mathematical problems does not appeal to me | | | --- | | --- | --- | --- | --- |
| **_** | The challenge of math problems does not appeal to me | | | --- | | --- | --- | --- | --- |
| **_** | Math puzzles are boring | | | --- | | --- | --- | --- | --- |
| **_** | I don’t understand how some people can spend so much time on math and seem to enjoy it | | | --- | | --- | --- | --- | --- |
| **-** | I would rather have someone give me the solution to a difficult math problem than have to work it out for myself | | | --- | | --- | --- | --- | --- |
| **_** | I do as a little work in math as possible | | | --- | | --- | --- | --- | --- |
| **Student’s Attitude Toward Problem-Based Learning (ATPBL)** | | | | | | | | | |
| **+** | I would prefer this approach (PBL) of solving mathematics problems than solving textbook problems. | | | --- | | --- | --- | --- | --- |
| **+** | This PBL approach makes mathematics problem solving more interesting and challenging. | | | --- | | --- | --- | --- | --- |
| **+** | Solving problems with a group of friends made the problem-solving process easier to manage. | | | --- | | --- | --- | --- | --- |
| **+** | I gained more confidence in solving mathematics problems through a PBL approach because of help from friends and teacher. | | | --- | | --- | --- | --- | --- |
| **+** | The challenge of solving the problem task kept me going and thinking. | | | --- | | --- | --- | --- | --- |
| **+** | I enjoyed working with my friends to solve Mathematics problems. | | | --- | | --- | --- | --- | --- |
