# Supplementary material for: Assessing the attitude and problem-based learning in mathematics through PLS-SEM modeling
Source: PLoS One. 2022 May 19;17(5):e0266363. doi: 10.1371/journal.pone.0266363 (PMC9119563; doi:10.1371/journal.pone.0266363)
Supplement: S2 Appendix — (DOCX) [file pone.0266363.s002.docx]

**Study on Assessing the Attitude and Problem-Based Learning in Mathematics in Secondary Schools in Punjab, Pakistan**

**(Teacher’s Questionnaire)**

| School | --------------------- | Gender | | --------------------- | | | |
| --- | --- | --- | --- | --- | --- | --- | --- |
| Qualification | --------------------- | Teaching Experience | | --------------------- | | | |
| **What are the effects of using Problem-based Learning (PBL) on student’s achievements?** Please rate them according to given scale.  SD= Strongly disagree, D= Disagree, U= Uncertain, A= Agree, SA= Strongly agree | | | | | | | |
| **Statements** | | | **Scale** | | | | |
| **Problem solving learning and students’ achievement** | | | **SD** | **D** | **U** | **A** | **SA** |
| You always get a good response from students are motivated actively to solve the problems by themselves. | | | --- | --- | --- | --- | --- |
| You find the problem-solving method supportive for learners of all abilities in the class. | | | --- | --- | --- | --- | --- |
| When I use this method, student achievement is high. | | | --- | --- | --- | --- | --- |
| Problem solving is helpful to eliminate cramming and last-minute revision. | | | --- | --- | --- | --- | --- |
| Problem solving is helpful to make a learner more skilled and confident. | | | --- | --- | --- | --- | --- |
| The mathematics curriculum is designed to use the problem-solving method frequently. | | | --- | --- | --- | --- | --- |
| Textbooks are structured to support problem solving strategies. | | | --- | --- | --- | --- | --- |
| Problem solving is helpful to improve students ‘performance in exams. | | | --- | --- | --- | --- | --- |
| **To determine the advantages in the adoption of a problem-solving approach in the learning of mathematics.** | | | | | | | |
| **Advantages of problem-solving learning** | | | **SD** | **D** | **U** | **A** | **SA** |
| Problem solving helps students to use mathematics in their daily life. | | | --- | --- | --- | --- | --- |
| Involvement and attention span of students can be enhanced. | | | --- | --- | --- | --- | --- |
| Understanding of the laws of mathematics helps develop induction and deduction skills. | | | --- | --- | --- | --- | --- |
| Students learn to draw diagram and pictures themselves to solve problems. | | | --- | --- | --- | --- | --- |
| Students are no longer afraid of long problem statements. | | | --- | --- | --- | --- | --- |
| Students find relief from panic near the examination. | | | --- | --- | --- | --- | --- |
| Problem solving reduces the need to revise prior to examinations. | | | --- | --- | --- | --- | --- |
| **How’s the teacher perception regarding/about Problem-Based Learning difficulties during class?** | | | | | | | |
| **Difficulties in using problem solving learning** | | | **SD** | **D** | **U** | **A** | **SA** |
| This method is difficult when students are larger in number in the classroom. | | | --- | --- | --- | --- | --- |
| This method is not suitable when time span is short for teaching. | | | --- | --- | --- | --- | --- |
| You spend more time to prepare a lesson. | | | --- | --- | --- | --- | --- |
| You need enough space, resources and feasible environment in the class. | | | --- | --- | --- | --- | --- |
| It is difficult to satisfy the learners of different abilities. | | | --- | --- | --- | --- | --- |
| It is more difficult to satisfy slow and weak learners through problem solving. | | | --- | --- | --- | --- | --- |
| The problem-solving method with IT requires extra training and is expensive. | | | --- | --- | --- | --- | --- |
