# Supplementary material for: Correction: Assessing the attitude and problem-based learning in mathematics through PLS-SEM modeling
Source: PLoS One. 2023 Jan 20;18(1):e0280909. doi: 10.1371/journal.pone.0280909 (PMC9858887; doi:10.1371/journal.pone.0280909)
Supplement: S2 File — (PDF) [file pone.0280909.s002.pdf]

PLOS ONE

RESEARCH ARTICLE

Assessing the attitude and problem-based learning in mathematics through PLS-SEM modeling

Samina Zamir, Zhang Yang*, Hao Wenwu, Uzma Sarwar

School of Education, Shaanxi Normal University, Xi'an, P.R. China

* zhangyang@snnu.edu.cn

Check for updates

OPEN ACCESS

Citation: Zamir S, Yang Z, Wenwu H, Sarwar U (2022) Assessing the attitude and problem-based learning in mathematics through PLS-SEM modeling. PLoS ONE 17(5): e0266363. https://doi.org/10.1371/journal.pone.0266363

Editor: Prabhat Mittal, Satyawati College (Eve.), University of Delhi, INDIA

Received: October 15, 2021

Accepted: March 18, 2022

Published: May 19, 2022

Copyright: © 2022 Zamir et al. This is an open access article distributed under the terms of the Creative Commons Attribution License, which permits unrestricted use, distribution, and reproduction in any medium, provided the original author and source are credited.

Data Availability Statement: All relevant data are within the manuscript and its Supporting Information files.

Funding: ZY received the grant for the Social Science Fund Project of Shaanxi Province "Legitimacy Analysis of Education and Training Market" (2019Q005). This study was also supported by the National Social Science Foundation of China Project (BAA170014).

Competing interests: The authors have declared that no competing interests exist.

Abstract

Mathematics plays a leading part in day-to-day life and has enhanced a necessary component for human accomplishments. Students from many countries do not reach the expected level in mathematics. Therefore, it is essential to pay close consideration to the causes related to ability in mathematics. Mathematics attitude is considered as one of the critical variables in the process of mathematics learning. This study aimed to determine students' attitudes and achievements through problem-based learning in mathematics. The selected study group contained 600 students and 35 teachers from rural public secondary schools in District Rawalpindi, Pakistan. The data collection was done using questionnaires from students and teachers and collected data analyzed by SPSS 23 and Amos 23. This study's result was carried out using Partial Least square structural equation Model (PLS-SEM), descriptive analysis, and hypotheses testing. The outcomes in this study indicated that the mean fluctuated between 1 to 4.5, 3.71 to 4.20, and Std. Deviation fluctuated between 0.6 to 2.0 and 0.75 to 1.55 in the students and teacher models, respectively. The results of the PLS-SEM students' model show a negative attitude towards mathematics. The teachers' PLS-SEM model showed the Effects of using problem-based learning (PBL) on students' achievements. According to the hypotheses testing, the acceptance of hypotheses by stating that the Confidence in Learning Mathematics Scale (C), Value of Mathematics Scale (V), and Student Mathematics Motivation Scale (M) are significant effects for the Students' Attitude Toward Problem-Based Learning (ATPBL). But the Attitude Toward Enjoyment in Mathematics Scale (AE) was rejected, and it did not significantly affect the ATPBL. As well as, the Problem-solving learning and students' achievement (PLA), Advantages of problem-solving learning (APL) and Difficulties in using problem-solving learning (DPL) have a significant positive effect on the ATPBL. Finally, this study suggested that teachers also adopt new teaching methods corresponding to mathematics, and there is a need to explore particular mathematics skills to enhance students' learning abilities.

1. Introduction

The most imperative things about societies are their learning ability, and learning is a necessary behaviour in life through genetic intelligence and the environment. There has been a lot

of advances in educational technology in the last few decades [1]. Learning ability constantly impact the human lifestyle. As well as it is, in both developed and developing economies, entrepreneurship is considered vibrant to the nation's competitive ability and a high-powered resource for decreasing regional inequities thereby allowing the development of the country [2,3]. According to that, the knowledge capabilities of an individual affect the student's way of life continually [4]. Scribbling, painting and drawing perform ana significant part in the growing up of children [5,6] and it is helping to build their knowledge, personality like that. Consequently, human societies attempt to increase the method of learning in education unceasingly. As well, the ability is the core dimension of personality between learning preferences and cognitive forms. It can be defined as the preferred personal method to collect and process information, decisions, interests, ideas, and attitudes [3].

Mathematics plays a dominant role in human lives, and it is broadly applied as an essential element in personal achievements and economics [7,8]. In the 21st century, mathematics plays a significant skill in individual satisfaction and involvement in society, school, and the labour market. It appears to be a key academic filter for students' educational trajectories [9]. Mathematics plays an essential role in supporting people to grow to reason, problem-solving skills, and thinking, and the importance of mathematics in the education system has gradually increased. Not only that, the impact of students' mathematics achievements, including students' ability, family socioeconomic status (SES), curriculum, many factors, peer influence, parental participation, school environment, and teachers' quality [10,11]. By taking a positive attitude towards mathematics, students will think that mathematics is fundamental, so they try to enhance their performance in mathematics [7]. However, learning mathematics has grown into a challenge for most students today. Lack of learning despair motivates many students to say, "I am not good at mathematics", even before trying to solve mathematical problems [11]. Hence, teachers have a significant part in enhancing students' mathematics achievement [10]. Emotional understanding, belief, and attitude are three major categories in the effective field of mathematics education [12].

However, recent worldwide determinations showed that students from many countries do not accomplish as anticipated in mathematics [13]. Therefore, one must pay close attention to the factors related to mastering mathematics and attitudes are one of the variables that can play a crucial role in learning mathematics [7].

The recent regeneration of mathematics education has brought new requirements. These provide students with meaningful activities that allow them to share their information in society. Various learning approaches focus on actions are used mainly in primary schools. One method is "problem-based learning" (PBL), which is a skill-based learning technique used to investigate and solve complicated real-life difficulties [14]. Most of the newest studies on PBL accentuate that it is a technique to enable students to enthusiastically play the part of learners. Most of the studies on PBL focus on teaching in different fields of education. These studies focus on mathematics education, science, engineering, and medicine [15]. Kaptan [16] documented that the PBL method is very important for students to improve the skills and knowledge learned in mathematics class to their daily issues and daily life. Theoretically, PBL is based on constructivism, and its instructional design method is based on problem-solving and "contextual learning" [14]. In general, PBL is considered to contribute to increasing and maintaining academic success [17] increase performance abilities [18] have a confident impact on attitudes towards classes [15], self-learning abilities and improve communication, as well as independent working abilities and motivation, and produce more reasonable explanations to problems [19]. Therefore, students' attitudes toward mathematics have been researching worldwide for several decades [20].

2. Theoretical reviews

Attitude is "a learned inclination on the part of an individual to respond positively or negatively to the concept, situation, some object, or another person" [21]. Thus, the attitude towards mathematics can be an aggregation of mathematical emotions and beliefs. Allport [22] defines an attitude as "a mental or neural state of readiness, prepared over practice, applying a directive or dynamic effect upon the individuals' feedback to all objects and circumstances with which it is associated". Adediwura [23] describes attitude as a persons' positive, neutral or negative thinking about mathematics. A positive attitude is very instructive because research shows that there is a link between student performance and their attitude toward mathematics [24]. Students who have a positive attitude toward mathematics have better problem-solving abilities and are better able to resolve unusual difficulties [25]. They capitalize more energy in solved problems and give up when the problem cannot be solved. Attitude is also be interchanged with personality and is recognized as a multidimensional structure, including self-confidence or anxiety, such as enjoyment or not, commitment or avoidance, beliefs about whether mathematics is difficult or easy, unimportant or important, uninteresting, interesting, and useless [12]. Köğce [26] showed that the mathematics attitude is subjective in some factors, and it can be considered as several groups: firstly, reasons connected with the student, secondly, reasons associated to the teacher and school, and finally reasons related to the society and environment. Reasons related to the students' mathematical results, their past practices [27], and social image of the mathematics. Not only that, but the reasons also related with the teachers and their content of knowledge, resources used in the classroom, the teaching methods, personality, teaching topics with real-life enriched examples [28], and the teachers' attitude towards mathematics. Therefore, their teachers' attitudes influence students' attitudes [29]; teachers' wrong beliefs about mathematics powerfully affect their teaching practices [30]. As well as it is vital to improving a positive attitude towards mathematics between students and teachers.

There have been a lot of improvements in educational technology in the last few decades [1] like online education. Students can use this technology any subject areas (especially mathematics) to improve their knowledge. But empirical studies have found that students feel that they learn better in physical classrooms than through online education [31]. Hence, Educational technology is affecting the students and teachers' attitude toward problem-based learning mathematics.

In recent times, many researchers have pointed out the student attitude and teachers' attitude towards problem-based learning in mathematics in several cities /countries around the world. The attitude towards mathematics has been considered for past years and shows a high relationship between attitude (including motivation, enjoyment, and self-confidence) and mathematical performance. Mezirow [32] defines learning as a cycle that starts from experience, continues to reflect, and leads to action, which becomes the experience of reflection. Valkenburg [33] found that children give their attention very rapidly to media content that was only moderately various from their existing capabilities and knowledge and teachers should give their attention for that [34]. Attitude towards mathematics is the students' and teachers' prepared preference to behave, perceive, feel, and think towards mathematics. Many studies have been established to assess the effect in mathematics [35].

Yılmaz [28] presented a positive and vital association between students' attitudes towards mathematics use and mathematics accomplishment. Secondly [36], proposed a progressive connection between mathematics accomplishment and mathematics attitudes. They revealed that scholars improve attitudes, ideas, and feelings about school subjects from different sources. Thirdly, Colomeischi [37] analyzed a correlation between learning style and gender,

attitude towards mathematics, and mathematical achievement. Thus, Bayaga [38] explained the students' attitudes toward mathematics achievement using a variety of factors (attitude, mathematics self-concept, school condition, family background, teaching, and parent's educational level) and approaches. We've looked into the relationship between math attitude and mathematics performance. A positive relationship between attitudes toward mathematics and academic achievement has been established in the majority of studies conducted across a range of age groups. According to some of the findings, having a negative attitude toward mathematics is associated with minor academic consequences in college students [39,40] and children [7]. However, in addition to doing so, Zsoldos-Marchis [24] investigated the problem-solving potential of various primary preschool teachers' attitudes toward mathematics.

Moreover, Russo [41] documented the association between math teachers' enjoyment and attitudes toward student struggle and the number of times teachers spent teaching math. There are more methods developed around the world to analyze attitudes towards mathematics. Among them, one of the most well-known analysis methods is the Partial Least Structural Equation Model (PLS-SEM), and it is a flexible modeling method without data distribution assumptions. It is also essential and suitable for various education analyses. The main aim of this study was to estimate the student attitude and teachers' attitudes towards problem-based learning in mathematics.

3. Methodology

3.1. Participants and data collection

The study population comprised 3,300 secondary mathematics students and 35 mathematics teachers in District Rawalpindi's 35 rural public secondary schools. The population is the mathematics students and teachers in North Punjab District Rawalpindi Government Areas as of the 2020/2021 academic session. This study selected the North Punjab district because the schools and education system are better than other areas. Moreover, belonging to the Rawalpindi district so it will be convenient to access the schools. This study selected rural schools because, in mathematics, 10th class students score low compared to urban schools.

First, purposive sampling will be used in identifying and selecting schools that meet the following criteria:

- Evidence of continuous presentation of candidates for external examination in mathematics.
- Availability of qualified mathematics teachers who used the problem-based learning method in their class.
- Availability of teacher's students and schools who agree to this study. Due to religious, cultural, regional, and local barriers.

The current study was approved by the Educational Research Ethics committee from the School of Education, Shaanxi Normal University. All procedures performed in the study involving human participants were in accordance with the ethical standards of the institutional research committee and consent was obtained from each respondent. Additional information regarding the ethical, cultural, and scientific considerations specific to inclusivity in global research is included in the Supporting Information (S1 Appendix).

By the above criteria, 35 schools will be purposively selected. In these schools, 600 mathematics (female, male) students and 35 mathematics teachers applied problem-based learning methods in their classes. The general overview of the students in the study is given in Tables 1 and 2 showed that the number of teachers in gender-wise and their qualifications.

Table 1. General characteristics of the students.

Gender	Age				Total
	13 years	14 years	15 years	16 years	
Girls	62	59	67	50	238
Boys	95	91	102	74	362
Total	157	150	169	124	600

https://doi.org/10.1371/journal.pone.0266363.t001

3.2. Data analysis

A structured questionnaire with a Likert scale was used to investigate the mathematics attitudes toward students (see S2 Appendix) and teachers (see S3 Appendix). Descriptive statistics, hypothesis testing is used for analysis. Analysis was performed by examining the correlations, covariance patterns between the observed measures and hypotheses testing were used for this study. There are seven (7) proposed hypotheses (H_1 to H_7) used for analysis (see Fig 1).

H_1: Confidence in Learning Mathematics Scale is positively influenced to Student's Attitude Toward Problem-Based Learning.

H_2: Value of Mathematics Scale is positively influence by Student's Attitude Toward Problem-Based Learning.

H_3: Attitude Toward Enjoyment in Mathematics Scale is positively influence to Student's Attitude Toward Problem-Based Learning.

H_4: Student Mathematics Motivation Scale is positively influencing to Student's Attitude Toward Problem-Based Learning.

H_5: Problem-solving learning and students' achievements positively influence Student's Attitude Toward Problem-Based Learning.

H_6: Difficulties in using problem-solving learning is positively influenced Student's Attitude Toward Problem-Based Learning.

H_7: Advantages of problem-solving learning is positively influenced to Student's Attitude Toward Problem-Based Learning.

Descriptive statistics are shown that provide a general overview of the data of the respondents. The collected data was analyzed by SPSS 23 version and Amos 23. For data analysis, Partial Least square structural equation Model (PLS-SEM) was used, interpreted in two stages. The first was to evaluate the student model, and the second was to assess the teachers' model. The first (Student's Attitude Toward Problem-Based Learning -ATPBL) model consisted of four constructs with 46 indicators—Confidence in Learning Mathematics Scale (C) = 12 indicators; Value of Mathematics Scale (V) = 12 indicators; Attitude Toward Enjoyment in

Table 2. General characteristics of the teachers.

Qualification	Male		Female	
	Frequency	Percent	Frequency	Percent
BS	3	9	5	14
M.Phil.	5	14	8	23
M.Sc.	6	17	8	23
Total	14	40	21	60

https://doi.org/10.1371/journal.pone.0266363.t002

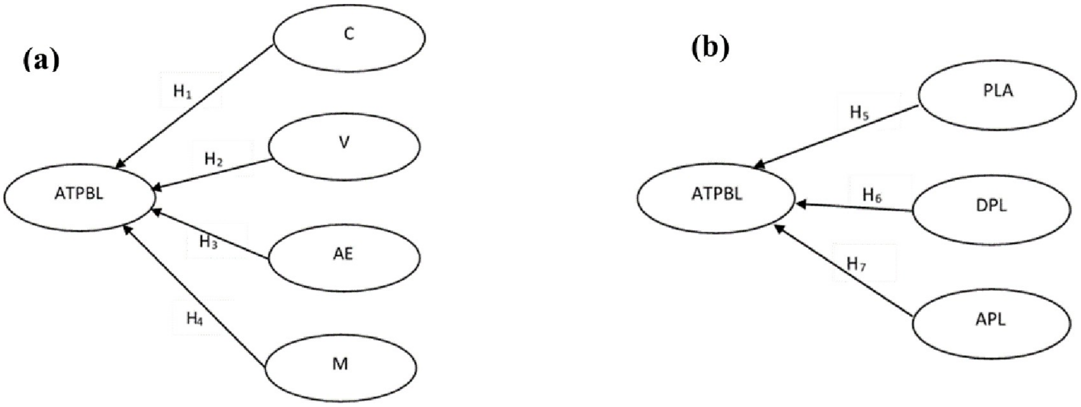

Fig 1. Theoretical model and hypotheses (a) students, (b) teachers.

https://doi.org/10.1371/journal.pone.0266363.g001

Mathematics Scale (AE) = 10 indicators; and Student Mathematics Motivation Scale (M) = 12 indicators. The second model consisted of three constructs with 22 indicators—Problem-solving learning and students' achievement (PLA) = 8 indicators, Advantages of problem-solving learning (APL) = 7 indicators, and Difficulties in using problem-solving learning (DPL) = 7 indicators can be seen in S4 Appendix.

4. Results and discussion

4.1. Student attitude towards problem-based learning in mathematics

According to Table 3, the mean fluctuated between 1 to 4.5 and Std. Deviation fluctuated between 0.6 to 2.0 and highly Std. Deviation reported from C4 (*I am always confused in my mathematics class.*) in the Confidence in Learning Mathematics Scale group. But the low value of Std. Deviation value reported from AE5 (*I really like mathematics*) in attitude toward enjoyment in mathematics scale group. Table 3 shows the results of descriptive statistics in the SEM model's exogenous variables.

According to Fig 2, the Confidence in Learning Mathematics Scale group had a high regression weight from C11 (In terms of my adult life, it is not important for me to do well in mathematics in high school). It recorded 0.664. But in the C1 (I have a lot of self-confidence when it comes to mathematics) showed that a low regression weight. It is recorded -22. As well as Confidence in Learning Mathematics Scale and Student's Attitude toward Problem-Based Learning presented the 0.11 Standardized Regression Weight. Value of Mathematics Scale (V) showed the high regression weight with V10 (Taking mathematics is a waste of time.), and it recorded 1.01. But in the V1 (Mathematics is a very worthwhile and necessary subject) showed a low regression weight. It is recorded -.11. As well as Value of Mathematics Scale and Student's Attitude toward Problem-Based Learning presented the 0.12 of Standardized Regression Weight. Hence, the Attitude toward Enjoyment in Mathematics Scale (AE) had a high regression weight from AE7 (Winning a prize in mathematics would make me feel unpleasantly conspicuous), and it recorded 0.88. Hence in the AE4 (I really like mathematics.) showed a low regression weight. It is recorded -.12.

As well as Attitude toward Enjoyment in Mathematics Scale and Student's Attitude toward Problem-Based Learning presented the 0.005 of Standardized Regression Weight. Furthermore, Student Mathematics Motivation Scale (M) had a high regression weight from M8 (The challenge of math problems does not appeal to me), and it recorded 0.90. Hence in the AE4 (I really like mathematics.) showed a low regression weight. It is recorded -.014. As well as Student Mathematics Motivation Scale and Student's Attitude toward Problem-Based Learning

Table 3. The results of descriptive statistics in the SEM model's exogenous variables.

Items	Mean	Std. Error of Mean	Std. Deviation	Variance
**C1**	3.828	0.052	1.267	1.605
**C2**	3.990	0.043	1.050	1.102
**C3**	3.925	0.055	1.349	1.819
**C4**	2.710	0.063	**1.544**	2.383
**C5**	4.270	0.037	0.912	0.832
**C6**	4.005	0.054	1.320	1.741
**C7**	3.542	0.056	1.379	1.901
**C8**	2.423	0.058	1.430	2.044
**C9**	2.152	0.057	1.391	1.935
**C10**	2.302	0.062	1.515	2.294
**C11**	2.527	0.061	1.494	2.233
**C12**	2.228	0.055	1.340	1.796
**V1**	3.788	0.053	1.299	1.686
**V2**	3.897	0.040	0.985	0.971
**V3**	3.913	0.056	1.360	1.849
**V4**	3.882	0.046	1.127	1.270
**V5**	4.183	0.037	0.895	0.801
**V6**	4.303	0.037	0.916	0.839
**V7**	4.033	0.037	0.911	0.830
**V8**	2.250	0.057	1.388	1.927
**V9**	3.800	0.050	1.228	1.509
**V10**	2.260	0.051	1.260	1.588
**V11**	1.997	0.046	1.126	1.269
**V12**	4.112	0.040	0.987	0.974
**AE1**	3.828	0.052	1.267	1.605
**AE2**	3.893	0.041	0.997	0.994
**AE3**	3.925	0.055	1.349	1.819
**AE4**	3.895	0.046	1.136	1.289
**AE5**	4.192	0.036	**0.889**	0.790
**AE6**	4.318	0.038	0.934	0.872
**AE7**	2.483	0.058	1.426	2.033
**AE8**	3.953	0.043	1.044	1.090
**AE9**	4.087	0.036	0.876	0.767
**AE10**	2.162	0.053	1.294	1.675
**M1**	3.788	0.040	0.984	0.968
**M2**	4.128	0.040	0.971	0.943
**M3**	2.363	0.056	1.370	1.878
**M4**	3.897	0.040	0.985	0.971
**M5**	3.913	0.056	1.360	1.849
**M6**	2.182	0.054	1.334	1.778
**M7**	2.555	0.060	1.476	2.177
**M8**	2.200	0.054	1.334	1.780
**M9**	2.138	0.047	1.152	1.328
**M10**	3.913	0.043	1.052	1.108
**M11**	2.545	0.059	1.438	2.068
**M12**	4.062	0.038	0.941	0.886
**ATPBL1**	3.780	0.040	0.987	0.973
**ATPBL2**	4.112	0.040	0.987	0.974
**ATPBL3**	3.788	0.040	0.984	0.968

(*Continued*)

Table 3. (Continued)

Items	Mean	Std. Error of Mean	Std. Deviation	Variance
**ATPBL4**	4.128	0.040	0.971	0.943
**ATPBL5**	3.788	0.053	1.299	1.686
**ATPBL6**	3.897	0.040	0.985	0.971

https://doi.org/10.1371/journal.pone.0266363.t003

presented 0.086 of Standardized Regression Weight. According to the SEM, the standardized estimation can be identified the most student have a negative attitude about mathematics. As well as Fig 2 showed that the squared multiple correlations (R^2). A strong positive correlation was reported in V10 (Taking mathematics is a waste of time) with a 1.0 value. V2 (I want to develop my mathematical skills), V12 (I expect to have little use for mathematics when I get out of school), M1 (I like math puzzles), M2 (Mathematics is enjoyable and stimulating to me), M4 (Once I start trying to work on a math puzzle, I find it hard to stop), M12 (I do as a little work in math as possible), C5 (I learn mathematics easily.), C12 (When I hear the word mathematics, I have a feeling of dislike.), AE1 (I have usually enjoyed studying mathematics in school.) showed that, the no correlation. Not only that, but there was also no negative correlation reported in this model.

4.2. Hypotheses testing for Student attitude towards problem-based learning in mathematics

The proposed hypotheses of this study were tested through the standardized coefficient values and p-values in AMOS 23.0. The students' model's dependent variable was the Student's

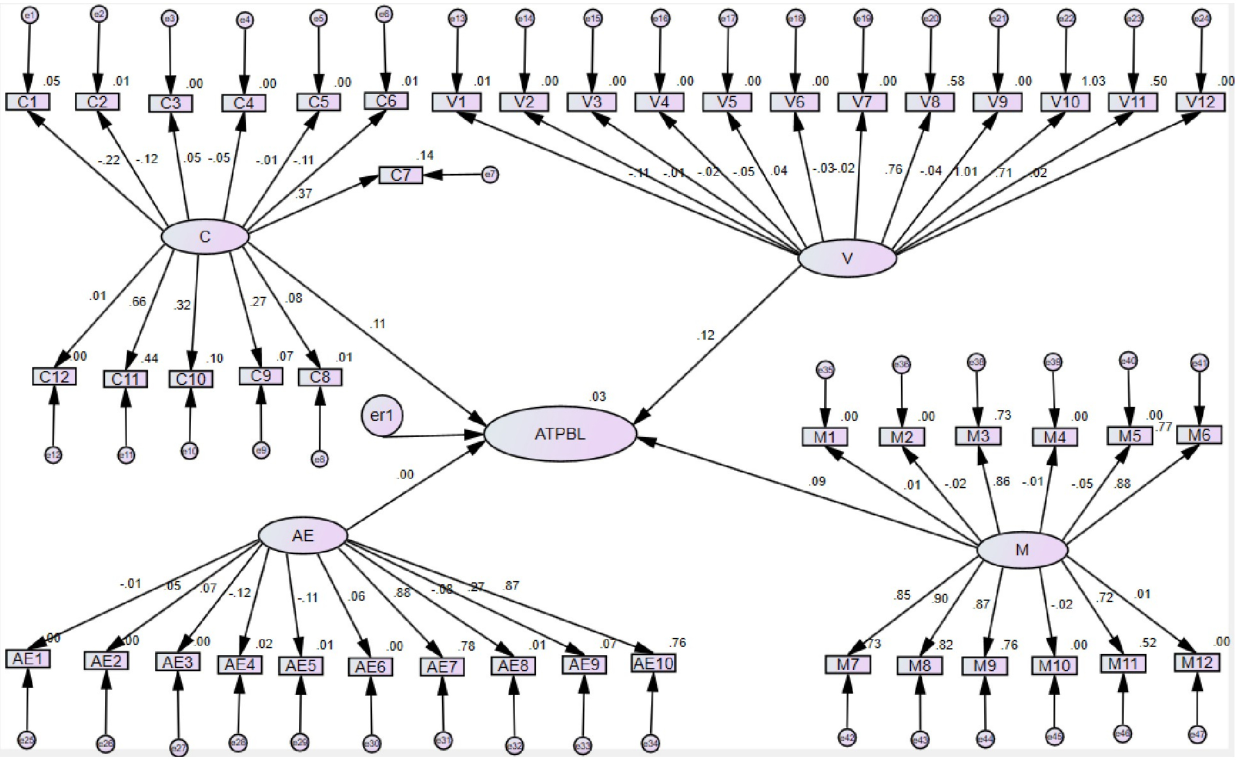

Fig 2. The results of structural model for PLS-SEM standardized estimation and hypotheses tests (students).

https://doi.org/10.1371/journal.pone.0266363.g002

Table 4. Hypotheses testing results.

Hypothesis	Hypotheses paths	Standard coefficients	P-values	Findings
H_1	C →ATPBL	0.11	0.001	Accept
H_2	V →ATPBL	0.12	0.001	Accept
H_3	AE → ATPBL	0.00	0.584	Reject
H_4	M → ATPBL	0.09	0.001	Accept

https://doi.org/10.1371/journal.pone.0266363.t004

Attitude Toward Problem-Based Learning (ATPBL). The Confidence in Learning Mathematics Scale (C), Value of Mathematics Scale (V), Attitude Toward Enjoyment in Mathematics Scale (AE), and Student Mathematics Motivation Scale (M) were independent variables.

Table 4 showed the acceptance of hypothesizes states that the Confidence in Learning Mathematics Scale (C), Value of Mathematics Scale (V), and Student Mathematics Motivation Scale (M) and these states are significant effects on the Student's Attitude Toward Problem-Based Learning. But hypotheses state that the Attitude Toward Enjoyment in Mathematics Scale (AE) was rejected, and it did not significantly impact the Student's Attitude Toward Problem-Based Learning (ATPBL).

4.3. Effects of using Problem-based Learning (PBL) on student's achievements

According to Table 5, the mean fluctuated between 3.71 to 4.20 and Std. Deviation fluctuated between 0.75 to 1.55 and high std. Deviation reported from PLA3 (*When I use this method, student achievement is high.*) in Problem solving learning and students' achievement group. But the low value of std. Deviation value reported from APL7 (*Problem-solving reduces the need to revise prior to examinations.*) in Advantages of the problem-solving learning group.

According to Fig 3, the problem-solving learning and students' achievement group had the high regression weight from PLA6 (*The mathematics curriculum is designed to use the problem-solving method frequently.*), and it recorded 0.97. However, the PLA1 (*You always get a good response from students who are motivated actively to solve the problems by themselves.)* showed a low regression weight. It is recorded -.269. As well as problem-solving learning and students' achievement (PLA) and Student's Attitude toward Problem-Based Learning presented the 0.106 Standardized Regression Weight. Advantages of problem-solving learning (APL) showed the high regression weight with APL7 (*Textbooks are structured to support problem-solving strategies*) and recorded 0.314. But in the APL1 (*Problem-solving helps students to use mathematics in their daily life.*) showed a low regression weight. It is recorded -.0.974. As well as Advantages of problem-solving learning (APL) and Student's Attitude toward Problem-Based Learning presented the 0.031 of Standardized Regression Weight. Hence, Difficulties in using problem-solving learning (DPL) had a high regression weight from DPL2 (*This method is not suitable when the time span is short for teaching.)*, and it recorded 0.94. Moreover, the DPL4 (You need enough space, resources, and feasible environment in the class.) showed the low regression weight. It is recorded -.153. As well as Difficulties in using problem-solving learning (DPL) and Student's Attitude toward Problem-Based Learning presented the 0.11 of Standardized Regression Weight.

As well as Fig 3 showed the squared multiple correlations (R^2) and strong positive correlation reported in APL1 (*You always get a good response from students are motivated actively to solve the problems by themselves.*), DPL2 (*This method is not suitable when time span is short for teaching.*), APL2 (*You find the problem-solving method supportive for learners of all abilities in the class.*), DPL6 (*It is more difficult to satisfy slow and weak learners through problem*

Table 5. Descriptive statistics' results in SEM model's exogenous variables.

Items	Mean	Std. Error of Mean	Std. Deviation	Variance
PLA1	4.20	0.18	1.05	1.11
PLA2	4.31	0.19	1.11	1.22
PLA3	4.06	0.26	**1.55**	2.41
PLA4	3.97	0.21	1.22	1.50
PLA5	4.17	0.18	1.07	1.15
PLA6	3.94	0.22	1.33	1.76
PLA7	3.97	0.21	1.22	1.50
PLA8	4.11	0.18	1.08	1.16
APL1	3.74	0.24	1.42	2.02
APL2	3.91	0.20	1.17	1.37
APL3	4.14	0.16	0.94	0.89
APL4	3.71	0.24	1.43	2.03
APL5	3.80	0.26	1.51	2.28
APL6	3.71	0.21	1.23	1.50
APL7	4.29	0.13	0.75	0.56
DPL1	3.91	0.16	0.95	0.90
DPL2	4.11	0.19	1.11	1.22
DPL3	3.97	0.15	0.89	0.79
DPL4	4.06	0.15	0.91	0.82
DPL5	3.94	0.17	1.03	1.06
DPL6	3.89	0.17	0.99	0.99
DPL7	4.06	0.16	0.94	0.88

https://doi.org/10.1371/journal.pone.0266363.t005

solving.), APL4 (*Students learn to draw diagram and pictures themselves to solve problems*.), with .949, .883, .883, .859, .844 .824, respectively. ATPBL (*Student's Attitude toward Problem-Based Learning*), APL3 (*When I use this method*, *student achievement is high*.) PLA5 (*Problem-solving is helpful to make a learner more skilled and confident.)* DPL1 (*This method is difficult when students are larger in number in the classroom*.) showed the very week but positive correlation. Not only that, but there was also no negative correlation reported in this model.

4.4. Hypotheses testing for effects of using Problem-based Learning (PBL) on student's achievements

The proposed hypotheses of this study were tested through the standardized coefficient values, and p-values in AMOS 23.0 for the teachers' model. In the teachers' model dependent variable was the Student's Attitude Toward Problem-Based Learning (ATPBL). The Problem-solving learning and students' achievement (PLA) Advantages of problem-solving learning (APL) and Difficulties in using problem-solving learning (DPL) were independent variables in this study.

Table 6 showed the acceptance of hypothesizes by stating that the Problem-solving learning and students' achievement (PLA), Advantages of problem-solving learning (APL), and Difficulties in using problem-solving learning (DPL). These states have a significant positive impact on the Student's Attitude Toward Problem-Based Learning (ATPBL).

5. Conclusion

Information about students' attitudes towards problem-based learning in mathematics is influential to both the students and the teachers [30]. The current study Partial Least Structural Equation Model (PLS-SEM) approach investigates the student attitude and teachers' attitude

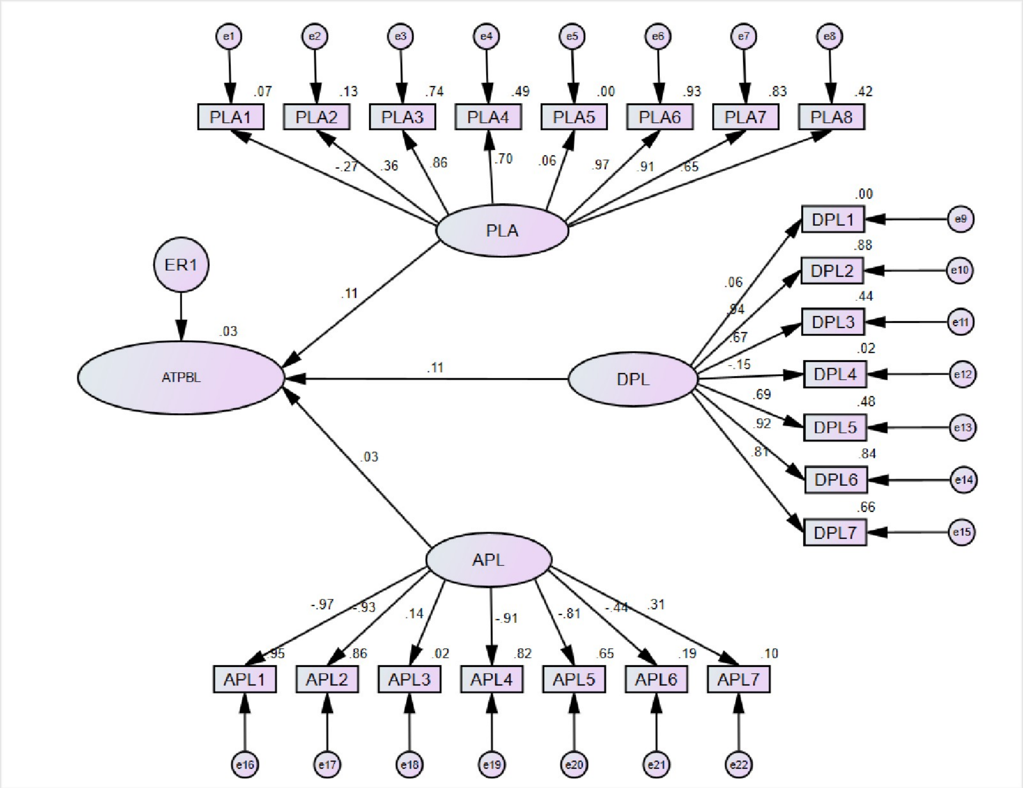

Fig 3. The Results of structural model for PLS-SEM standardized estimation and hypotheses tests (teachers).

https://doi.org/10.1371/journal.pone.0266363.g003

towards problem-based learning in mathematics. The demographic data of this study have also exposed those 600 students and 36 teachers are competent in handling mathematics.

In firstly, this study estimated the student attitude towards problem-based learning in mathematics. The PLS-SEM model showed that the mean fluctuated between 1 to 4.5 and Std. Deviation fluctuated between 0.6 to 2.0. Among the 46 indicators, the C4 (I am always confused in my mathematics class) showed a high Std. Deviation and, but the low value of Std. Deviation value reported from AE5 (I really like mathematics) indicator.

According to the regression weight, in the students' model, the high weight record in C11 (In terms of my adult life it is not important for me to do well in mathematics in high school.) and it recorded 0.664, V10 (Taking mathematics is a waste of time.). It recorded 1.01, AE7 (Winning a prize in mathematics would make me feel unpleasantly conspicuous) and it recorded 0.88, M8 (The challenge of math problems does not appeal to me), and it recorded 0.90.

According to the regression weight, in the teachers' model, the high weight record PLA6 (The mathematics curriculum is designed to use the problem-solving method frequently) and

Table 6. Hypotheses testing results.

Hypothesis	Hypotheses paths	Standard coefficients	P-values	Findings
H_5	PLA →ATPBL	0.11	0.001	Accept
H_6	DPL→ ATPBL	0.11	0.001	Accept
H_7	APL →ATPBL	0.03	0.001	Accept

https://doi.org/10.1371/journal.pone.0266363.t006

is recorded 0.97. APL7 (Textbooks are structured to support problem-solving strategies.), and it recorded 0.314. DPL2 (This method is not suitable when time span is short for teaching.), and it recorded 0.94.

According to the hypothesizes testing, the acceptance of hypothesizes by stating that the Confidence in Learning Mathematics Scale (C), Value of Mathematics Scale (V), and Student Mathematics Motivation Scale (M) and these states are significant effects on the Students' Attitude Toward Problem-Based Learning. But it hypothesizes by stating that the Attitude Toward Enjoyment in Mathematics Scale (AE) was rejected, and it did not significantly affect the Students' Attitude Toward Problem-Based Learning (ATPBL). As well as the acceptance of hypothesizes by stating that the Problem-solving learning and students' achievement (PLA), Advantages of problem-solving learning (APL) and Difficulties in using problem-solving learning (DPL) has a significant positive impact on the Students' Attitude Toward Problem-Based Learning (ATPBL).

This study significantly revealed students' attitudes towards mathematics and the attitudes of teachers who use it to teach mathematics. Finally, this study suggested that teachers should also adopt new teaching methods corresponding to mathematics. There is a need to explore particular mathematics skills to enhance students' learning abilities.

Supporting information

S1 Appendix. This appendix contains Inclusivity in global research.
(DOCX)

S2 Appendix. This appendix contains students' questionnaire.
(DOCX)

S3 Appendix. This appendix contains teachers' questionnaire.
(XLSX)

S4 Appendix. This appendix contains variables.
(DOCX)

S1 Dataset.
(RAR)

Acknowledgments

The first author would like to thank his parents who support him in this work. We thank those anonymous reviewers whose comments/suggestions helped to improve and clarify this manuscript.

Institutional review board statement

This study is approved by the Educational Research Ethics committee from the School of Education, Shaanxi Normal University. All procedures performed in the study involving human participants were in accordance with the ethical standards of the institutional research committee.

Informed consent statement

Informed consent was obtained from all subjects involved in the study.

Author Contributions

Conceptualization: Samina Zamir.

Data curation: Samina Zamir, Uzma Sarwar.

Formal analysis: Samina Zamir.

Funding acquisition: Zhang Yang.

Investigation: Zhang Yang.

Methodology: Samina Zamir.

Project administration: Zhang Yang, Hao Wenwu.

Resources: Zhang Yang.

Software: Uzma Sarwar.

Supervision: Zhang Yang, Hao Wenwu.

Validation: Samina Zamir.

Writing – original draft: Samina Zamir, Uzma Sarwar.

Writing – review & editing: Zhang Yang, Hao Wenwu.

References

1. Chakraborty P, Mittal P, Gupta M S, Yadav S, Arora A. Opinion of students on online education during the COVID-19 pandemic. Human Behavior and Emerging Technologies. 2021; 3 (3): 357–365. https://doi.org/10.1002/hbe2.240.
2. Mittal P, Raghuvaran S. Entrepreneurship education and employability skills: the mediating role of e-learning courses. Entrepreneurship Education. 2021; 4 (2):153–167. https://doi.org/10.1007/s41959-021-00048-6.
3. Mittal P. Big data and analytics: a data management perspective in public administration. International Journal of Big Data Management. 2020; 1(2):152. https://doi.org/10.1504/ijbdm.2020.112415.
4. Orhun N. An investigation into the mathematics achievement and attitude towards mathematics with respect to learning style according to gender. International Journal of Mathematical Education in Science and Technology. 2007; 38(3):321–33. https://doi.org/10.1080/00207390601116060.
5. Brooks M. Drawing, visualization and young children's exploration of "big ideas". International Journal of Science Education, 2009. 31(3):319–341. https://doi.org/10.1080/09500690802595771.
6. Yadav S, Chakraborty P, & Mittal P. (2021). Designing Drawing Apps for Children: Artistic and Technological Factors. International Journal of Human-Computer Interaction. 2021. 1–15. https://doi.org/10.1080/10447318.2021.1926113.
7. Geary DC. International differences in mathematical achievement: Their nature, causes, and consequences. Current Directions in Psychological Science. 1996; 5(5):133–7. https://doi.org/10.1111/1467-8721.ep11512344.
8. Primi C, Bacherini A, Beccari C, Donati MA. Assessing math attitude through the Attitude Toward Mathematics Inventory–Short form in introductory statistics course students. Studies in Educational Evaluation. 2020; 64:100838. https://doi.org/10.1016/j.stueduc.2020.100838.
9. Pitsia V, Biggart A, Karakolidis A. The role of students' self-beliefs, motivation and attitudes in predicting mathematics achievement: A multilevel analysis of the Programme for International Student Assessment data. Learning and Individual Differences. 2017; 55:163–73.
10. Hattie J. Visible learning: A synthesis of over 800 meta-analyses relating to achievement. Routledge; 2008.
11. Yu C, Li X, Wang S, Zhang W. Teacher autonomy support reduces adolescent anxiety and depression: An 18-month longitudinal study. Journal of adolescence. 2016; 49:115–23 https://doi.org/10.1016/j.adolescence.2016.03.001 PMID: 27042976
12. McLeod DB. Research on affect in mathematics education: A reconceptualization. Handbook of research on mathematics teaching and learning. 1992; 1:575–96.

13. Fleischman HL, Hopstock PJ, Pelczar MP, Shelley BE. Highlights from PISA 2009: Performance of US 15-Year-Old Students in Reading, Mathematics, and Science Literacy in an International Context. NCES 2011–004. National Center for Education Statistics. 2010.
14. Uygun N, Tertemiz NI. Effects of problem-based learning on student attitudes, achievement and retention of learning in math course. Egitim ve Bilim. 2014; 39(174).
15. Nardone CF, Lee RG. Critical inquiry across the disciplines: Strategies for student-generated problem posing. College Teaching. 2010; 59(1):13–22.
16. Kaptan F, Korkmaz H. Fen eğitiminde probleme dayalı öğrenme yaklaşımı. Hacettepe Üniversitesi Eğitim Fakültesi Dergisi. 2001; 20(20).
17. Demirel M, Turan B. The effects of problem based learning on achievement, attitude, metacognitive awareness and motivation. HACETTEPE UNIVERSITESI EGITIM FAKULTESI DERGISI-HACETTEPE UNIVERSITY JOURNAL OF EDUCATION. 2010; (38):55–66.
18. Araz G, Sungur S. Effectiveness of problem-based learning on academic performance in genetics. Biochemistry and Molecular Biology Education. 2007; 35(6):448–51. https://doi.org/10.1002/bmb.97 PMID: 21591143
19. Diggs LL. Student attitude toward and achievement in science in a problem-based learning educational experience. University of Missouri-Columbia; 1997.
20. Hodges CB, Kim C. Improving college students' attitudes toward mathematics. TechTrends. 2013; 57 (4):59–66. https://doi.org/10.1007/s11528-013-0679-4.
21. Aiken LR Jr. Attitudes toward mathematics. Review of educational research. 1970; 40(4):551–96.
22. Allport G W. Attitudes. In Murchison C. (Ed.), A handbook of social psychology. Worcester, MA: Clark University Press. 1995. https://doi.org/10.2466/pms.1995.80.3c.1187 PMID: 7478876
23. Adediwura AA. The Development and Confirmatory Factor Analysis of a Scale for the Measurement of Gifted Students Attitude towards Mathematics. World Journal of Education. 2011; 1 (1):52–62. https://doi.org/10.5430/wje.v1n1p52.
24. Zsoldos-Marchis I. Changing pre-service primary-school teachers' attitude towards Mathematics by collaborative problem solving. Procedia-Social and Behavioral Sciences. 2015; 186:174–82. https://doi.org/10.1016/j.sbspro.2015.04.100.
25. Marchiş I. Relation between students' attitude towards mathematics and their problem solving skills. PedActa. 2013; 3 (2):59–66.
26. Köğce D, Yıldız C, Aydın M, Altındağ R. Examining elementary school students' attitudes towards mathematics in terms of some variables. Procedia-Social and Behavioral Sciences. 2009; 1 (1):291–5.
27. Maio GR, Haddock G, Verplanken B. The psychology of attitudes and attitude change. Sage. 2018.
28. Yılmaz Ç, Altun SA, Olkun S. Factors affecting students' attidude towards Math: ABC theory and its reflection on practice. Procedia-Social and Behavioral Sciences. 2010; 2(2):4502–6.
29. Ford MI. Teachers' beliefs about mathematical problem solving in the elementary school. School Science and Mathematics. 1994; 94 (6):314–22.
30. Uusimaki L, Nason R. Causes Underlying Pre-Service Teachers' Negative Beliefs and Anxieties about Mathematics. International Group for the Psychology of Mathematics Education. 2004; 369–376.
31. Bojović Ž, Bojović PD, Vujošević D, Šuh J. Education in times of crisis: Rapid transition to distance learning. Computer Applications in Engineering Education. 2020; 28(6):1467–89.
32. Mezirow J. A critical theory of adult learning and education. Adult Education Quarterly.1981; 3–24.
33. Valkenburg PM, Vroone M. Developmental changes in infants' and toddlers' attention to television entertainment. Communication Research. 2004; 31(3):288–311.
34. Yadav S, Chakraborty P, Mittal P, Arora U. Children aged 6–24 months like to watch YouTube videos but could not learn anything from them. Acta Paediatrica, International Journal of Pediatrics. 2018; 107 (8): 1461–1466. https://doi.org/10.1111/apa.14291 PMID: 29558569
35. Chamberlin SA. A review of instruments created to assess affect in mathematics. Journal of Mathematics education. 2010; 3(1):167–82.
36. Khatoon T, Mahmood S. Mathematics anxiety among secondary school students in India and its relationship to achievement in mathematics. European Journal of Social Sciences. 2010; 16(1):75–86.
37. Colomeischi AA, Colomeischi T. The students 'emotional life and their attitude toward mathematics learning. Procedia-Social and Behavioral Sciences. 2015; 180:744–50. https://doi.org/10.1016/j.sbspro.2015.02.192.
38. Bayaga A, Wadesango N. Analysis of students' attitudes on mathematics achievement-factor structure approach. International Journal of Educational Sciences. 2014; 6(1):45–50. https://doi.org/10.1080/09751122.2014.11890116.

39. Chen L, Bae SR, Battista C, Qin S, Chen T, Evans TM, Menon V. Positive attitude toward math supports early academic success: Behavioral evidence and neurocognitive mechanisms. Psychological Science. 2018; 29(3):390–402. https://doi.org/10.1177/0956797617735528 PMID: 29364780

40. Niepel C, Burrus J, Greiff S, Lipnevich AA, Brenneman MW, Roberts RD. Students' beliefs and attitudes toward mathematics across time: A longitudinal examination of the theory of planned behavior. Learning and Individual Differences. 2018; 63:24–33. https://doi.org/10.1016/j.lindif.2018.02.010.

41. Russo J, Bobis J, Sullivan P, Downton A, Livy S, McCormick M, Hughes S. Exploring the relationship between teacher enjoyment of mathematics, their attitudes towards student struggle and instructional time amongst early years primary teachers. Teaching and Teacher Education. 2020; 88:102983. https://doi.org/10.1016/j.tate.2019.102983.
